# Short chain fatty acids delay the development of hepatocellular carcinoma in HBx transgenic mice

**DOI:** 10.1016/j.neo.2021.04.004

**Published:** 2021-05-01

**Authors:** Noreen McBrearty, Alla Arzumanyan, Eugene Bichenkov, Salim Merali, Carmen Merali, Mark Feitelson

**Affiliations:** a Department of Biology, College of Science and Technology, Philadelphia, PA, USA; b Department of Pharmaceutical Sciences, School of Pharmacy, Temple University, Philadelphia, PA, USA

**Keywords:** Proteomics, Ras signaling, Chronic liver disease, Pathogenesis, Hepatitis B x

## Abstract

Chronic infection with hepatitis B virus (HBV) is a major risk factor for the development of hepatocellular carcinoma (HCC). The HBV encoded oncoprotein, HBx, alters the expression of host genes and the activity of multiple signal transduction pathways that contribute to the pathogenesis of HCC by multiple mechanisms independent of HBV replication. However, it is not clear which pathways are the most relevant therapeutic targets in hepatocarcinogenesis. Short chain fatty acids (SCFAs) have strong anti-inflammatory and anti-neoplastic properties, suggesting that they may block the progression of chronic liver disease (CLD) to HCC, thereby identifying the mechanisms relevant to HCC development. This hypothesis was tested in HBx transgenic (HBxTg) mice fed SCFAs. Groups of HBxTg mice were fed with SCFAs or vehicle from 6 to 9 months of age and then assessed for dysplasia, and from 9 to 12 months of age and then assessed for HCC. Livers from 12 month old mice were then analyzed for changes in gene expression by mass spectrometry-based proteomics. SCFA-fed mice had significantly fewer dysplastic and HCC nodules compared to controls at 9 and 12 months, respectively. Pathway analysis of SCFA-fed mice showed down-regulation of signaling pathways altered by HBx in human CLD and HCC, including those involved in inflammation, phosphatidylinositol 3-kinase, epidermal growth factor, and Ras. SCFA treatment promoted increased expression of the tumor suppressor, disabled homolog 2 (DAB2). DAB2 depresses Ras pathway activity, which is constitutively activated by HBx. SCFAs also reduced cell viability in HBx-transfected cell lines in a dose-dependent manner while the viability of primary human hepatocytes was unaffected. These unique findings demonstrate that SCFAs delay the pathogenesis of CLD and development of HCC, and provide insight into some of the underlying mechanisms that are relevant to pathogenesis in that they are responsive to treatment.

Liver cancer is the sixth most commonly diagnosed and second most lethal cancer worldwide [1]. Hepatocellular carcinoma (HCC) accounts for about 80% of global primary liver cancer diagnoses [1]. The incidence of HCC continues to increase, with rates tripling in the United States over the past twenty years [2]. Chronic infection with hepatitis B virus (HBV) is a major risk factor for HCC. HBV has infected roughly 2 billion people worldwide, and among these, an estimated 250 million become carriers who are at increased risk for the development of hepatitis, cirrhosis, and HCC [3]. In the United States, the 2-year survival rate from the time of diagnosis is less than 50%, and 5 year survival rate is only 8.9%, highlighting the urgent need for more effective therapeutic options [4], [5], [6].

HBV encoded hepatitis B x antigen (HBx) contributes centrally to the development of HCC by a variety of putative mechanisms, but it is not clear which of these mechanisms are critical for tumor development [3,7]. Recurrent cycles of cell death and regeneration in chronic liver disease (CLD) are associated with increased integration of the HBx gene into host DNA, and production of functional HBx [8] leading to alterations of host gene expression [7], chromosomal instability [8], and altered signaling pathways crucial to cell survival, inflammation, angiogenesis, and immune responses [8]. Among the signaling pathways altered by HBx, phosphatidylinositol 3-kinase (PI3K), Ras, and nuclear factor kappa-B (NF-κB) promote cell survival and growth [9]. Aberrant activation of other pathways involving platelet-derived growth factor and vascular endothelial growth factor (VEGF) by HBx have also been implicated in HCC initiation and progression [9]. HBx alters host gene expression through epigenetic regulation, by stimulating histone deacetylases (HDACs) and DNA methyltransferases. HBx is capable of silencing tumor suppressors and activating host oncogenes to promote carcinogenesis [10]. However, therapeutic modalities targeting HBx and/or its related pathways, and which pathways are most relevant to the pathogenesis of HCC, have not been developed.

Patients with HCC have alterations in the composition of their gut microbiome [11], suggesting corresponding changes in the levels and ratios of pro- and anti-inflammatory metabolites in the gut. Among these, short chain fatty acids (SCFAs) are produced by the anaerobic fermentation of dietary fiber carried out by gut microorganisms. SCFAs regulate cell growth and differentiation, prevent inflammation, inhibit cell proliferation and induce apoptosis in cancer cells [12,13]. SCFAs appear to oppose the actions of HBx on many of the same molecules and pathways that HBx exploits in carcinogenesis. For example, SCFAs reduce cell proliferation by inhibiting histone deacetylases [14], whereas HBx stimulates the activity of selected HDACs [10], suggesting that SCFAs may reverse some of the epigenetic effects of HBx on chromatin structure [8,15]. SCFAs inhibit pro-inflammatory NF-κB signaling, and have been shown to be anti-inflammatory in a variety of chronic diseases [14,16], while HBx stimulates NF-ĸB [17] and associated inflammation [18]. Importantly, HBx expression and activity is increased in an oxidative environment characteristic of inflammation [19]. Further, the intensity and distribution of intrahepatic HBx expression correlates with the severity of CLD [20], suggesting that a chronic inflammatory environment potentiates the actions of HBx. In this context, SCFAs have been used to delay the onset of colitis-associated colorectal cancer [21] which develops on a background of chronic inflammation, as does HCC from CLD. Thus, experiments were designed to test the hypothesis that SCFAs delayed the development of dysplastic nodules and/or HCC in HBx transgenic (HBxTg) mice that closely recapitulates many steps in the pathogenesis of CLD and HCC seen in human HBV carriers [22]. Given that there are no effective treatments for patients with CLD, and that HCC is diagnosed too late in most cases for the application of curative therapies, the results of this study introduces a new approach consisting of a simple, effective therapeutic intervention strategy which delays the progression of CLD and the development of HCC by blocking pathways that contribute to disease pathogenesis and cancer hallmarks.

## Materials and methods

### Short chain fatty acids (SCFAs)

SCFAs were purchased from Sigma (St. Louis, Mo.). These consist of the sodium salts of butyrate (202410), propionate (P1880) and acetate (S2889).

### Mice and treatments

HBxTg mice (C57Bl/6 × DBA) were created, as previously described [23], and further modified [22]. In these mice, hepatitis and steatosis develops in animals by 6 months of age, dysplasia by 9 months, and HCC nodules by 12 months [22]. Sibling littermates consisting of 6- and 9-month old mice of both genders, were treated 5 days per week during daylight hours with SCFAs or phosphate buffered saline (PBS) by oral gavage for 3 months. Concentrations of each SCFA were 40 mM of butyrate, 67.5 mM of acetate, and 25.9 mM of propionate as reported previously [24]. After 3 months of treatment, livers were removed and then examined histologically. Tumor diameters were measured using a caliper. Liver samples from three mice in the 12-month old group were prepared for proteomics (see below). Additional details related to treatment are outlined in the Supporting Information. All animal protocols were conducted according to the NIH guide for the care and use of Laboratory animals (NIH Publications No. 8023, revised 1978) and approved by the Temple University Institutional Animal Care and Use Committee.

### Immunohistochemistry

Immunohistochemistry was performed using paraffin-embedded sections of tissue samples and are detailed in the Supporting Information. Primary antibodies were anti-HBx (anti-99) [25], as well as rabbit polyclonal antibodies against disabled homolog 2 (DAB2) (ab76253 Abcam, Cambridge, MA) and the leucine-rich repeat protein Shoc2 (ab106430 Abcam).

### Sodium dodecyl sulfate/polyacrylamide gel electrophoresis and western blotting

Protein lysates were prepared from snap frozen liver tissue from 12-month old control and SCFA-treated HBxTg mouse liver samples as outlined in Supporting Information.

### RAS Activity assay

A Ras activation assay (ab211158, Abcam, Branford, CT) was performed on control and SCFA-treated HBxTg mouse liver samples to isolate GTP bound Ras according to manufacturer's instructions as outlined in Supporting Information.

### Cell culture

Huh7 and Hep3B cells were stably transduced with HBx gene by recombinant retroviruses (referred to as Huh7x and Hep3Bx, respectively) and cultured without the selection of individual clones as previously described [26]. Primary human hepatocytes were purchased from Zen-Bio, Inc. (catalog no. HP-F, Research Triangle Park, North Carolina) and cultured according to manufacturer's instructions.

### Cell viability and treatment

Cells were plated in 96 well plates in complete DMEM and incubated in 5% CO_2_ overnight. Treatment consisted of different concentrations of SCFAs (0, 1, 5, and 10 mM) for 24 hours. Cell survival was then determined in triplicate using the MTS assay (G3582, Promega, Madison, WI) according to manufacturer's instructions.

### Proteomics and data analysis

Liver tissues from 12-month old control (n = 3) and SCFA-treated (n = 3) HBxTg mice were homogenized and extracted proteins were digested. Peptides were acidified and loaded onto an activated in-house-made cation stage tip, purified and eluted into 3 fractions [27]. Mass spec analysis was performed on these fractions as previously described [28]. A detailed description of the procedure is in the Supporting Information. Differentially altered proteins were identified from corresponding peptides by Maxquant and Andromeda software and then organized into functional pathways using panther software (version 15.0) [29].

### Statistics

All data were analyzed using excel or graphpad software and further statistical analyses were performed as outlined in the Supporting Information.

For proteomics, a t-test was performed on quantified proteins on select proteins with low variance within a group and on proteins that were differentially expressed to statistical significance between groups. Those proteins that were differentially expressed (greater than 2-fold difference in magnitude compared with control mice or *P* < 0.05) were selected for Panther pathway analysis. Additionally, those proteins that were detected in the majority or all samples from one group and no samples from the comparison group were selected for pathway analysis and literature searches, as reported previously [30].

## Results

### SCFA treatment reduced the number of dysplastic and HCC nodules

**Fig. 1:**
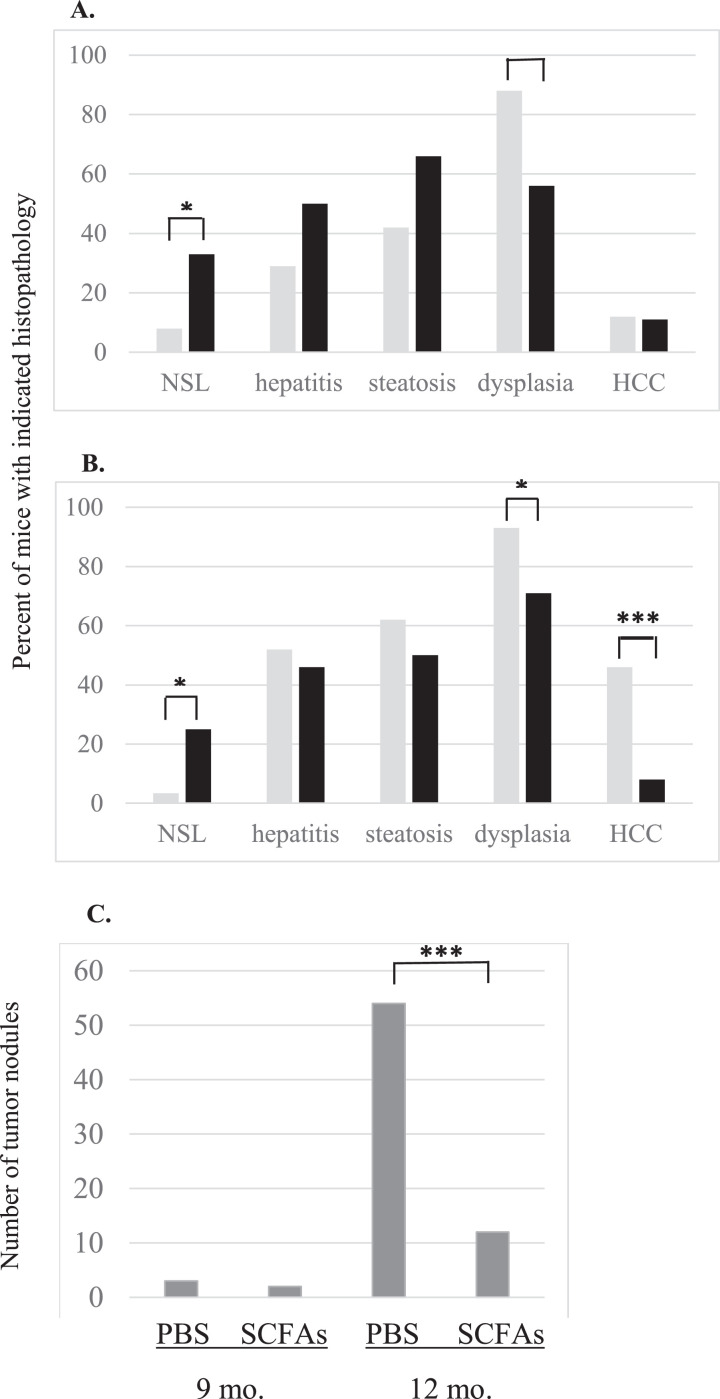
Impact of SCFAs upon the pathogenesis of chronic liver disease. Mice in the 9 month old group (A) and 12 month old group (B) were fed PBS (gray bars) or short chain fatty acids (black bars) for three months prior to euthanasia, and the histopathology was scored by light microscopy. Results are reported as the percentage of mice in each group with the corresponding histopathological lesions. (C) Number of visible tumor nodules identified in mice euthanized at 9 months or 12 months of age treated with PBS or SCFAs. *P < 0.05, **P < 0.02, ***P < 0.001.

**Fig. 2:**
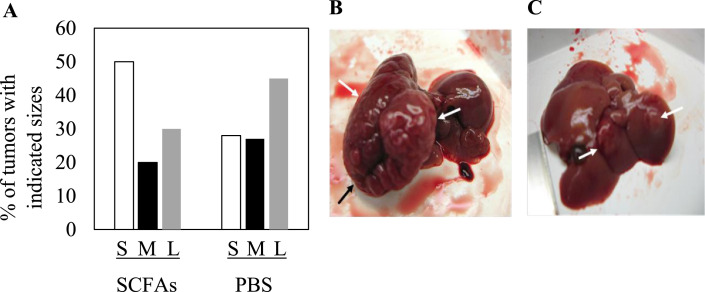
Impact of SCFA treatment upon tumorigenesis. (A) Percentage of tumor nodules from 12 month old mice treated with SCFAs or with PBS relative to tumor size. S: small tumors (<0.5 cm), M: medium size tumors (0.5–1 cm), L: large tumors (>1 cm). (B) Example of a large tumor from a PBS treated mouse. (C) Example of 2 small tumors from a SCFA treated mouse. Arrows in (B) and (C) denote the position of the tumor nodules.

To determine the impact of SCFAs on the development of dysplastic nodules, HBxTg mice were treated with SCFAs or PBS from 6 to 9 months of age (henceforth referred to as ‘9-month group’). To determine the impact of SCFAs upon HCC development, an additional group of mice was treated with SCFAs or PBS from 9 to 12 months of age (henceforth referred to as ‘12-month group’). At the end of treatment, livers from 9- and 12-month groups were evaluated for histopathology and 12-month group livers further analyzed by proteomics. There was no statistical difference in the number of mice that developed hepatitis or steatosis in SCFA treated compared to PBS control mice in either age group (Fig. 1A and B), although this was expected since the mice developed these disease stages before treatment was initiated. However, a greater number of mice in each age group treated with SCFAs had normal liver histology compared to PSS controls (Fig. 1A and B, *P* < 0.05). In contrast, significantly fewer SCFA treated mice developed dysplasia in both the 9-month (*P* < 0.02) and 12-month groups (*P* < 0.05; Fig. 1A and B). Further, SCFAs reduced the number of mice that developed HCC compared to control mice in the 12-month group (Fig. 1B, *P* < 0.001). Among mice that developed tumors in the 12 month old group, SCFA treated mice had 54 visible tumor nodules compared to 12 in the control mice (Fig. 1C, *P* < 0.001). In addition, SCFA treated mice in this group had predominantly small sized tumors compared to predominately large tumors that developed in PBS treated mice (Fig. 2, *P* < 0.001). When liver sections from 9 and 12 month old mice were examined for histopathology by light microscopy in sections from five different blocks from each lobe, HCC was present in the livers of mice that also had other lesions characteristic of CLD (data not shown), as in human carriers with HCC. Thus, although HCC develops on a background of CLD in both treated and control mice, SCFAs delay the pathogenesis of HBV-associated HCC.

### SCFAs specifically reduce cancer cell viability

**Fig. 3:**
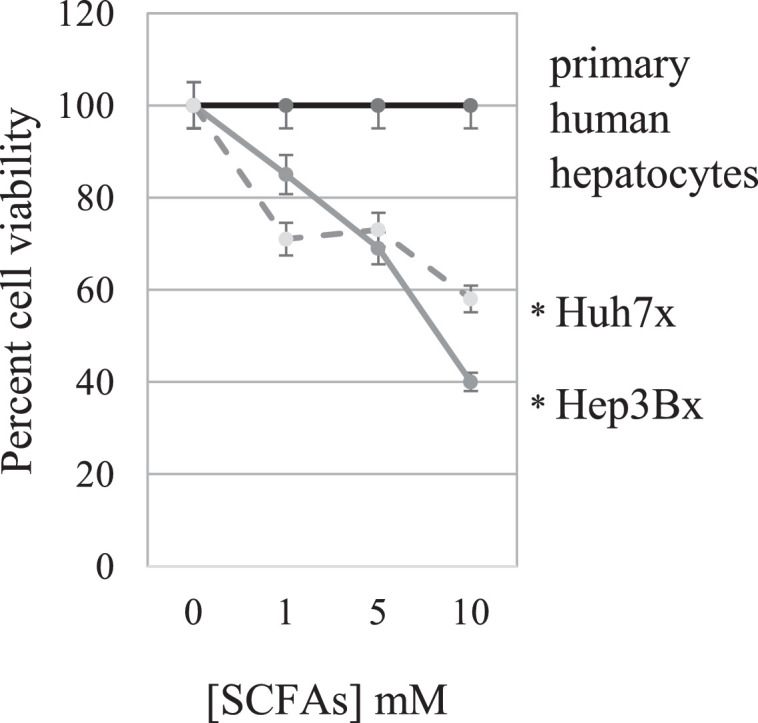
Effect of SCFAs on primary human hepatocytes and two HBx expressing human HCC cell lines. Primary human hepatocytes and the HCC cell lines transfected with HBx (Hep3Bx and Huh7x) were treated with increasing concentrations of SCFAs and assessed for cell viability using MTS assay. All measurements were performed in triplicate. Results are expressed as the percent viability of SCFA treated compared to PBS treated cells. *P < 0.01.

Since SCFAs were able to delay tumor development in HBxTg mice, experiments were designed to evaluate whether SCFAs had an impact on human HCC cell viability. When previously described Hep3Bx and Huh7x human hepatoma cell lines constitutively expressing HBx [26] were treated with SCFAs, cell viability decreased in a dose-dependent manner over 24 hours. In contrast, the viability of primary human hepatocytes was unaffected by SCFA treatment at the same doses and time-period (*P* < 0.01; Fig. 3). These observations were consistent with the in vivo experiments, demonstrating one way that SCFA treatment partially inhibited tumor growth.

### Differential expression of proteins by proteomics

Mass spectrometry-based proteomics was performed on SCFA-treated and control 12-month old HBxTg mouse livers to determine the effect of SCFAs on protein expression in biological processes and signaling pathways at the age when tumors appear. Accordingly, 3 biological replicates were included in each group analyzed. Tissue from different lobes of the liver were taken from each sample. Although these may have included microscopic tumors, the vast majority of the cells in these samples were nontumor. Among the more than 3000 proteins identified, 222 proteins were differentially expressed in the 12-month group. Differentially expressed proteins include those expressed 2-fold or greater from one another with a *P* < 0.05 as well as proteins detected in the majority or all samples in one group and not detected in any samples of the comparison group (Table 1). The mass spectrometer used in this study, Q exactive, is capable of detecting proteins present in as little as 1 ng of sample [31].

**Table 1:** Differentially expressed proteins associated with biological processes that are altered by SCFAs compared to PBS in the 12 month old liver.

Biological process	Protein name	Fold Change Treated/Control
	STE20-like serine/threonine-protein kinase	2.37
	Probable ATP-dependent RNA helicase DDX47	4.47
	Disabled homolog 2 (DAB2)	4.68
	Cyclin-dependent-like kinase 5	0.14
Apoptosis	Programmed cell death protein 10	0.22
	Dual serine/threonine and tyrosine protein kinase	4.28
	Baculoviral IAP repeat-containing protein 6	4.33
	Nck-associated protein 1	0.23
	Beta-catenin-like protein 1	0.14
	Protein prune homolog	0.23
	Centromere/kinetochore protein zw10 homolog	0.22
	Cyclin-dependent-like kinase 5	0.14
	DCC-interacting protein 13-alpha	0.23
Cell cycle	Baculoviral IAP repeat-containing protein 6	4.33
	BRISC complex subunit Abro1	4.82
	Growth arrest and DNA damage-(GADD) inducible proteins-interacting protein 1	0.21
	Charged multivesicular body protein 1b-1	0.21
Dephosphorylation	Haloacid dehalogenase-like hydrolase domain-containing protein 2	0.94
	Dual specificity protein phosphatase 23	0.15
DNA repair	Actin-like protein 6A	4.72
Electron transport,	Cytochrome c oxidase subunit 1	4.46
respiratory chain	Cytochrome b-c1 complex subunit 8	0.92
	Protein SCO2 homolog, mitochondrial	0.21
Endocytosis	Disabled homolog 2	4.68
Ion transport	V-type proton ATPase catalytic subunit A	0.96
Immunity, Innate	Chitinase domain-containing protein 1	0.22
immunity	Complement C5	0.22
	Protein jagunal homolog 1	0.22
	Choline/ethanolamine phosphotransferase 1	4.48
Lipid biosynthesis	CDP-diacylglycerol- 3-phosphatidyltransferase	4.83
	Phosphatidate cytidylyltransferase, mitochondrial	0.22
Mitochondrial transport	ATP-binding cassette sub-family B member 10, mitochondrial	0.22
	28S ribosomal protein S18b, mitochondrial	4.89
	28S ribosomal protein S7, mitochondrial	0.13
Mitochondrial	39S ribosomal protein L38, mitochondrial	0.22
Translation	39S ribosomal protein L3, mitochondrial	0.23
	39S ribosomal protein L53, mitochondrial	0.22
	28S ribosomal protein S7, mitochondrial	0.13
mRNA processing	Crooked neck-like protein 1	4.97
	RNA-binding protein with serine-rich domain 1	0.21
Neutrophil degranulation	N-acetylgalactosamine-6-sulfatase	0.22
Peroxisome biosynthesis	Peroxisomal membrane protein PEX16	0.22
Prenylated protein catabolism	Prenylcysteine oxidase	0.93
Protein biosynthesis	ATP-dependent RNA helicase Dhx29	4.62
	Mitochondrial import receptor subunit TOM20 homolog	4.92
	ELKS/Rab6-interacting/CAST family member 1	0.13
	Transcription and mRNA export factor ENY2	4.33
	Nuclear pore complex protein Nup98	0.19
	Importin subunit alpha-1	0.23
	Importin-5	0.22
Protein transport	Exportin-7	0.14
	Golgi SNAP receptor complex member 2	0.14
	Exocyst complex component 5	0.23
	Exocyst complex component 2	0.23
	Protein jagunal homolog 1	0.22
	Protein MON2 homolog	0.14
	WASH complex subunit strumpellin	0.23
	Charged multivesicular body protein 1b-1	0.21
	Ribosomal protein S6 kinase beta-2	0.24
Signal	Protein S100-A11	0.22
transduction	Shoc2	0.24
	MEK2	0.22
	Transcription elongation factor A protein 3	4.70
	Beta-arrestin-1	0.23
	Transcription and mRNA export factor ENY2	4.33
	SWI/SNF complex subunit SMARCC2	4.71
Transcription	Actin-like protein 6A	4.72
regulation	Transcription initiation factor TFIID subunit 5	0.22
	Cryptochrome-1	0.22
	Leucine-rich repeat flightless-interacting protein 1	4.55
	Nucleoplasmin-3	4.52
Translation	Protein quaking	0.23
regulation	Heterogeneous nuclear ribonucleoprotein L-like	7.48
Transport	Aquaporin-9	0.21
	Microtubule-associated proteins 1A/1B light chain 3B	0.23
	Ubiquitin carboxyl-terminal hydrolase 19	0.24
	Ubiquitin-conjugating enzyme E2 J1	0.14
Ubiquitin-	E3 ubiquitin-protein ligase ZNRF2	0.24
Conjugation	Ubiquitin fusion degradation protein 1 homolog	0.96
	Baculoviral IAP repeat-containing protein 6	4.33
	BRISC complex subunit Abro1	4.82
	Proteasome inhibitor PI31 subunit	0.21
	NHL repeat-containing protein 3	0.22
Unfolded protein response	Derlin-2	4.45
	Vacuolar fusion protein CCZ1 homolog	4.14
Vesicle-mediated transport	Dynactin subunit 4	0.15
	Centromere/kinetochore protein zw10 homolog	0.22

**Fig. 4:**
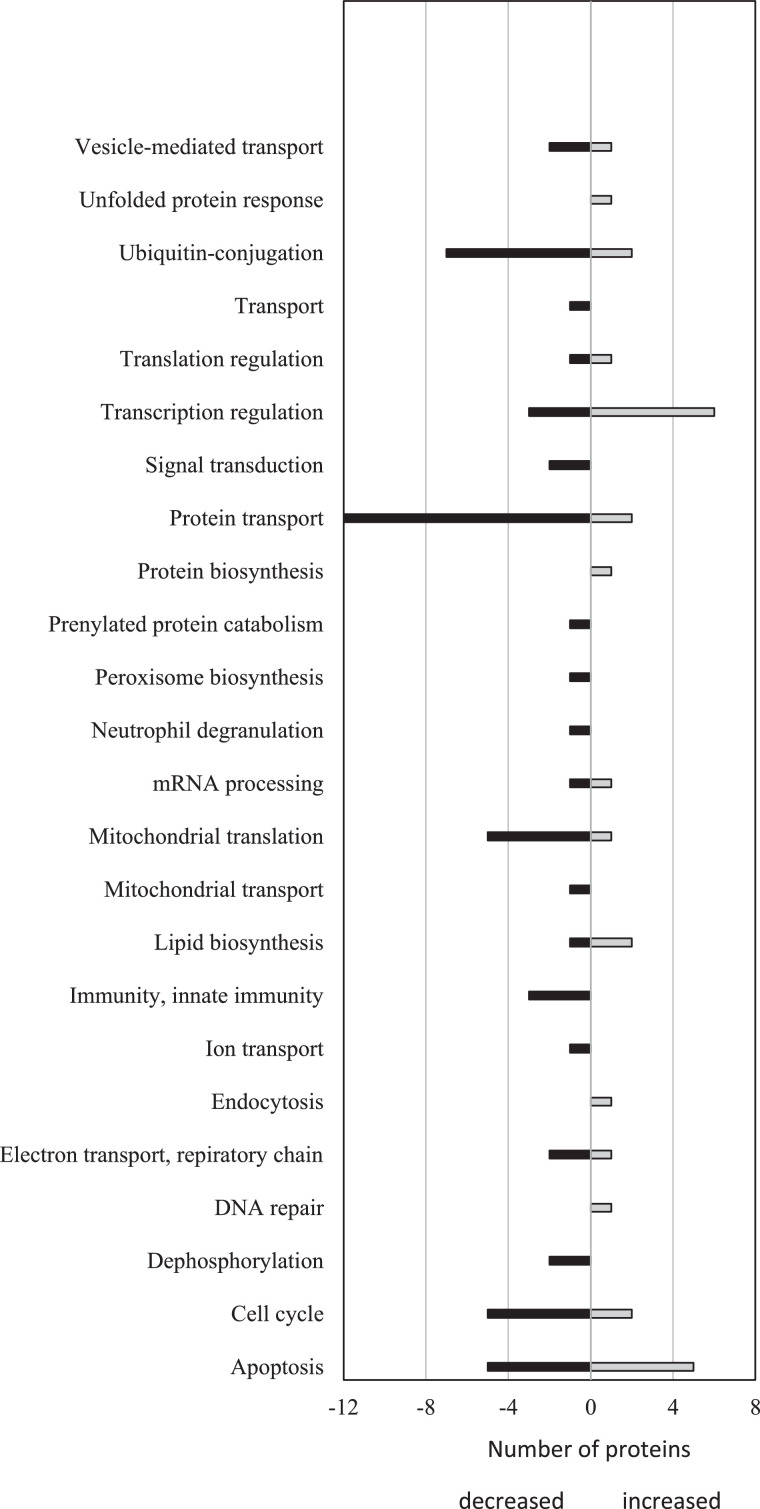
Biological processes altered by SCFAs. Number of proteins with decreased (black bars) or increased (gray bars) expression in 12-month old livers from HBxTg mice after SCFA treatment compared to PBS controls arranged by GO biological processes.

Differentially expressed proteins in livers of SCFA-treated compared to PBS-treated mice were arranged by their GO biological processes (Fig. 4, Table 1). Pathway analysis of 12-month old livers showed that SCFA treatment was associated with the downregulation of pathways known to be activated by HBx in hepatocarcinogenesis. These pathways include inflammation, PI3K, platelet-derived growth factor, fibroblast growth factor, insulin-like growth factor, epidermal growth factor, Wnt, VEGF, and Ras (Fig. 4, Table 1). Proteins associated with these pathways were up-regulated in PBS fed livers but undetectable in SCFA livers. Aberrant activation of these signaling pathways have all been implicated in HCC initiation and progression [9,32,33]. These pathways drive cell proliferation and growth, block apoptosis, and promote angiogenesis, all of which are important to the pathogenesis of HCC.

Identification of differentially expressed proteins by discovery mass spectroscopy permitted their grouping into a number of distinct functional subsets. Among the fourteen differentially expressed genes that mediate protein transport, thirteen of them were detectable in control livers, but were below the limit of detection in the SCFA treated samples, suggesting that SCFAs down-regulate protein transport (Table 1). These include down-regulated expression of proteins involved in trafficking between the Golgi and endosome (MON), a protein regulating exocytosis (exocyst complex components 2 and 5), nuclear import and export (importin-5, exportin-7, importin subunit α-1, nuclear pore complex protein Nup98), and NF-κB signaling (ELKS/Rab6 interacting CAST family member 1). Although there are ten proteins involved in apoptotic pathways that are differentially expressed after SCFA treatment, it is not clear whether SCFAs promotes apoptosis (via increased expression of STE20-like ser/thr kinase, ATP-dependent RNA helicase DDX47, and the tumor suppressor DAB2), or protects from apoptosis (via up-regulation of the baculoviral IAP repeat-containing protein 6, down regulation of β-catenin-like protein 1 and the tumor suppressor protein prune homolog). In transcriptional regulation, SCFAs are known transcriptional regulators via histone deacetylase inhibition, and treatment results in the up-regulated expression of transcription elongation factor A protein 3, ENY2, actin-like protein 6A, and leucine-rich repeat flightless-interacting protein 1 (a transcriptional repressor), as well as down-regulated expression of β-arrestin-1, TFIID subunit 5, and cryptochrome-1. Transcription may also be altered by differential expression of the chromatin remodeling proteins SWI/SNF complex subunit of SMARCC2 and nucleoplasmin-3. The expression of 6 mitochondrial proteins are also altered, being detectable in PBS control livers but undetectable in the livers of SCFA-treated mice. The expression of smaller numbers of proteins are also altered in a variety of other pathways (Fig. 4, Table 1) by SCFAs, but their biological significance remains to be explored.

List of differentially expressed proteins and corresponding *P* values grouped according to their related biological functions.

### Validation of differentially expressed DAB2 and downstream signaling proteins

**Fig. 5:**
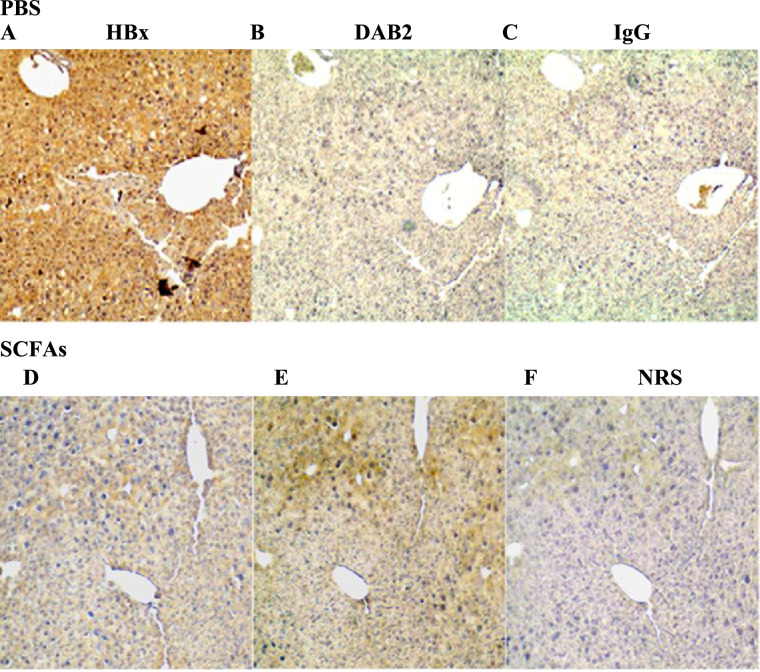
Effect of SCFAs upon the intrahepatic expression of HBx and DAB2. Immunohistochemical staining for HBx (A and D), DAB2 (B and E), normal IgG (D) or normal rabbit serum (NRS) (F) in 12 month old mouse livers from animals treated with PBS (A-C) or SCFAs (D-F). The results shown here are representative of stained samples from 20 control mice and 19 SCFA-treated mice (x40).

The results above showed that SCFA treatment was associated with a decrease in the size and appearance of HCC nodules in some mice, and smaller tumors in others (Fig. 2). Further work was carried out to validate one of these altered pathways that may contribute to these differences. SCFA treatment resulted in the up-regulated expression and/or stabilization of the tumor suppressor, DAB2 (Table 1), which suppresses Ras signaling. Verification of DAB2 identity was provided by mass spectrometry spectrum analysis with quantitation by Maxquant and Andromeda software (Fig. S1). The identities of the leucine-rich repeat protein Shoc2 and mitogen-activated protein kinase kinase 2, downstream effectors of Ras signaling, were also verified by spectral analysis (Fig. S1). Given that Ras signaling is activated in 50-100% of human HCCs [32], and is also activated by HBx [33], SCFA up-regulation of DAB2 may partially block the activity of HBx. When immunohistochemistry was performed for HBx, many of the SCFA and placebo treated mice showed diffuse, lobular and scattered tissue staining in the nuclear and cytoplasmic compartments of cells, although the differences were not statistically significant (data not shown). However, the intensity of HBx staining decreased in SCFA treated mice compared to controls (compare Fig. 5A and D, *P* < 0.02). Cytoplasmic staining was observed in liver samples evaluated from all mice, while half the animals also had nuclear HBx. When consecutive tissue slides from these same mice were stained for DAB2, scattered single cells showed weak cytoplasmic and sometimes nuclear staining for DAB2 in both SCFA and placebo treated mice, but these differences were not statistically significant (data not shown). However, DAB2 staining was more widespread in a larger number of scattered cells and lobular tissue staining was observed in SCFA treated compared to control tissues (compare Fig. 5B and E, *P* < 0.025). Staining specificity was demonstrated using normal rabbit immunoglobulin G (Fig. 5C) or normal rabbit serum (Fig. 5F). Thus, although staining was not uniform within a tissue section (Fig. 5) and between tissue samples obtained from different mice (data not shown), all showed an inverse correlation between HBx and DAB2 staining that was treatment dependent.

**Fig. 6:**
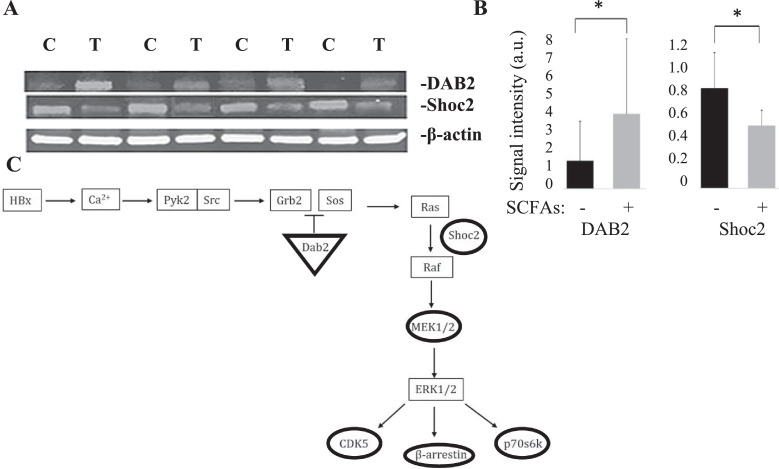
Validation of SCFA mediated inhibition of Ras signaling. (A) Representative western blot of DAB2 and Shoc2 from treated compared to control livers. (B) Summary of differentially expressed DAB2 and Shoc2 from treated (n = 12) compared to control (n = 12) mice (*P < 0.01). Signal density was measured in arbitrary units. (C) Summary of proteomics and western blot data of SCFAs on Ras-related proteins in 12-month old HBxTg mouse livers. Proteins with increased expression after SCFA treatment are indicated by a triangle, and proteins decreased by SCFA treatment are indicated by circles. Proteins in the Ras pathway that were not differentially expressed by SCFA treatment are indicated by a box.

An important property of discovery mass spectroscopy is the limited sensitivity in the detection of low abundance peptides, and the stringent cut-off values for Maxquant and Andromeda. Thus, western blotting was used to confirm the observations above, and to identify other proteins downstream of DAB2 that were also down-regulated by SCFA treatment. The results showed that DAB2 was up-regulated 2.6-fold in SCFA treated compared to control mice, and that for Shoc2 (Fig. 6A and B), which is required for the Ras to activate Ref. [34], the inverse was observed (Fig. 6A and B; *P* < 0.01). These observations suggest that up-regulation of DAB2 should also result in suppressed levels of other down-stream effectors, such as mitogen activated protein kinase kinase 1/2, cyclin dependent kinase 5, β-arrestin1, and the ribosomal kinase S6 kinase beta-2, all of which were highly expressed in the livers of PBS treated mice but undetectable in mice treated with SCFAs in the proteomics analysis (Fig. 6C). The finding of detectable DAB2 in western blots of PBS treated mice, and the differential expression of Shoc2 demonstrated by western blot but not by discovery based mass spectroscopy in SCFA treated mice, reflects the generally higher sensitivity of antibody detection in western blotting relative to mass spectroscopy. These combined observations suggest that differential expression of DAB2 contributes importantly to Ras signaling in HCC development, and is responsive to SCFA treatment in that the latter restores DAB2 expression in 12 month old livers with delayed tumor onset and growth.

**Fig. 7:**
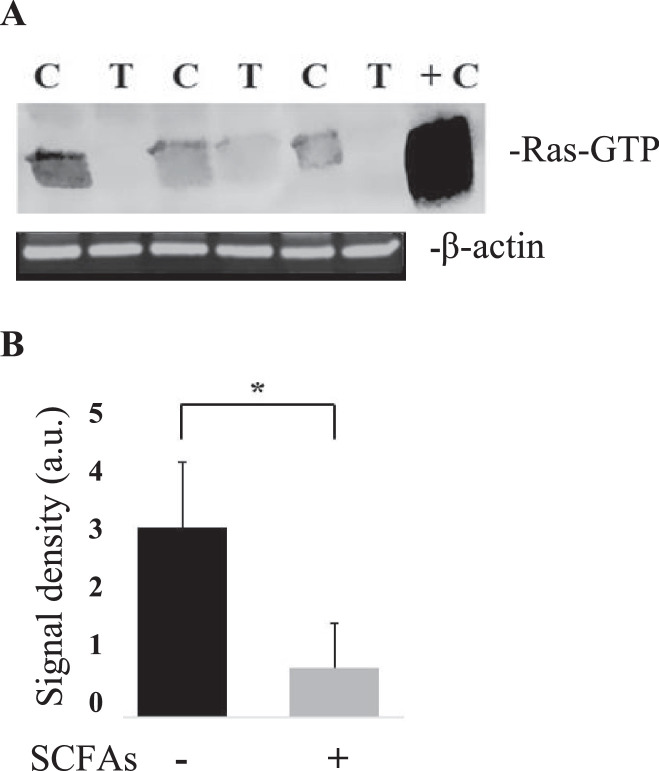
SCFA modulation of Ras activity. (A) Pulldown assay for activated Ras in three mice treated as controls [C] with PBS. Another three mice were treated with SCFAs [T] prior to analysis. +C = positive control. (B) Summary of Ras pulldown in seven mice treated with PBS compared to another seven treated with SCFAs *P < 0.001. Signal density was measured in arbitrary units (a.u.).

If DAB2 acts through Ras signaling, then there should be an inverse correlation between DAB2 expression levels and Ras activity. Ras is a small GTPase that cycles between its active, GTP-bound form, and its inactive, GDP-bound form. Accordingly, experiments were designed to determine the levels of the active form of Ras (Ras-GTP) in the 12-month old SCFA and PBS fed livers using a glutathione S-transferase-pulldown assay. SCFA-treated HBxTg mouse livers showed a 4-fold decrease in expression of Ras-GTP compared to control samples (Fig. 7), demonstrating that SCFA treatment is associated with decreased levels of active Ras (*P* < 0.001), but no changes in the levels of total Ras protein, as determined by proteomics (Table 1) and western blotting (data not shown). Given that Ras stimulates many processes important to carcinogenesis, and is known to be activated in HCC, these results suggest that the ability of SCFAs to promote DAB2 expression suppresses Ras activity, and that this suppression contributes to the delayed pathogenesis of HCC in this animal model.

## Discussion

Experiments were designed to identify changes occurring in the liver of HBxTg mice at the time when HCC nodules develop. SCFAs significantly reduced the number of mice that developed dysplasia and HCC (Fig. 1). Unexpectedly, the incidence of steatosis was higher in both treated groups compared to controls by 9 months of age, although these differences were not statistically significant. This may be due to a higher intake of fatty acids in the treated group, as previously reported [35]. However, there was a significant drop in the percent of mice that developed dysplasia by 9 months, suggesting that SCFAs attenuate disease progression (Fig. 1A). Among the 12 month group treated with SCFAs, there was also a significant drop in dysplasia, but a very strong drop in the percent of mice that developed HCC, suggesting that SCFAs delay or may prevent the progression of CLD to HCC (Fig. 1B). At 12 months, SCFA treated mice also had far fewer (Fig. 1C) and smaller tumors (Fig. 2) than control mice. The latter was confirmed in vitro, in which SCFAs inhibited the growth of the HBx positive human HCC cell lines, Huh7x and Hep3Bx (Fig. 3). These findings extend prior observations showing that butyrate inhibits the proliferation of HepG2.2.15 cells [36]. Butyrate may also delay or prevent tumor progression by promoting differentiation of hepatoma cell lines [37], although further studies are needed to establish whether SCFAs slow tumor progression in vivo. Importantly, treatment of primary human hepatocytes with SCFAs resulted in no discernable loss of viability (Fig. 3), suggesting that pathways effecting the viability of tumor cells were much more sensitive to SCFAs than the same pathways in normal hepatocytes, and/or that SCFAs are metabolized differently in normal compared to tumor cells. In addition, there is a statistically significant increase in the percentage of SCFA treated mice at both 9 and 12 months with normal liver histology compared to controls, but it is not clear whether this indicates CLD regression or whether CLD never develops. Either way, SCFAs have a major impact upon disease pathogenesis.

To distinguish the nature of these changes in signaling pathways and patterns of gene expression that underlie the effects of SCFAs, proteomics was conducted in multiple liver samples harvested from 12-month old mice. The results showed differential expression of many proteins in a variety of biological processes (Fig. 4), underscoring the pleiotropic effects of SCFAs upon multistep carcinogenesis. Pathway analysis of these differentially expressed proteins in the liver revealed suppressed Ras, PI3K, VEGF, transforming growth factor-beta, interferon signaling, and inflammation associated pathways, among others in response to SCFA treatment (Table 1). These pathways are known to be activated by HBx [9,[38], [39], [40]]. In addition, NF-κB, which is constitutively activated by HBx [17], and contributes importantly to the pathogenesis of HCC [18], is epigenetically down-regulated by SCFAs, especially butyrate [11]. Among the downregulated cancer-related pathways herein, Ras, PI3K, VEGF, fibroblast growth factor, and epidermal growth factor are all activated by HBx in HCC, and all crosstalk with NF-κB, suggesting that NF-κB inhibition may also occur. HBx protects infected cells from apoptosis by promoting activity of the PI3K and Ras pathways [7,32]. This deregulation was found to promote HCC progression through unrestrained cell proliferation, invasion, and metastasis. Alternations in other pathways involving angiogenesis (VEGF signaling), cell death (apoptosis signaling), immune mediated destruction (T cell activity), sustained proliferative signaling (insulin-like growth factor, Ras and platelet-derived growth factor), tumor promoting inflammation (chemokine and cytokine signaling), as well as invasion and metastasis (integrin signaling), all of which are down-regulated by SCFAs, are hallmarks of cancer that may underlie the delay in the multiple steps that ultimately result in the development of HCC.

Previously, HBx was shown to synergize with Kras to promote the formation and progression of HCC [41]. This relationship also led to the deregulated expression and activity of Akt, transforming growth factor-beta, and β-catenin, among other proteins [41]. Proteomic analysis of HBV-infected tissue confirmed HBx increases oxidative stress through interaction with HIF-1α [42], a pathway that was also demonstrated to be altered by the proteomic analysis herein. Further analysis of HBV-HCC tissue compared to adjacent non-tumor tissue showed altered expression of β-catenin-related proteins, NF-κB signaling components, ribosomal subunits, ubiquitin-related proteins, respiratory complex and metabolism-related protein [43], corroborating changes also detected in the present study (Table 1, Fig. 4). This work uniquely establishes that some of the proteins and pathways altered by HBx in the pathogenesis of HCC are reversed by treatment with SCFAs.

Treatment with the multi-kinase inhibitor, sorafenib, and related compounds, has been the standard of care for patients with late HCC. Importantly, the inhibition of the PI3K, platelet-derived growth factor, VEGF, insulin-like growth factor, transforming growth factor-beta, and Ras pathways by sorafenib is largely regarded as responsible for the drug's chemotherapeutic effect [44]. One of the problems limiting the efficacy of sorafenib may be that treatment is provided to patients with advanced HCC, and if many of the critical changes leading to cancer already occur prior to the development of tumors, as suggested herein by analysis of 12 month old livers (and not tumors), earlier intervention may have prophylactic value. Remarkably, SCFAs had no effect on the viability of normal hepatocytes (Fig. 3), demonstrating that they are specifically toxic to malignant cells, and that they may provide an approach for limiting toxicity among HBV carriers who already have significant liver damage.

Given that SCFAs reduce the incidence of HCC in 12-month old treated livers, and that the Ras signaling pathway is both stimulated by HBx and is commonly activated in HCC [9,32,33], additional work was carried out to validate that the activity and differential expression of Ras pathway components. The results in Figs. 5 and 6 show increased expression of the tumor suppressor, DAB2 and decreased expression of Shoc2, a downstream Ras signaling pathway component. Ras activity was also significantly diminished by SCFA treatment (Fig. 7). Importantly, epigenetic silencing of DAB2 in HCC has been associated with Ras activation, resulting in HCC initiation and progression [45]. Since DAB2 negatively regulates Ras by competing with son of sevenless homolog binding to growth factor receptor-bound protein 2, disrupting the formation of the latter complex, and suppressing consequent activation of Ras [46], may be one mechanism whereby SCFAs delay the pathogenesis of HCC. Activation of this pathway has also been found in several other human cancers including breast, colon, and prostate cancer, suggesting that SCFAs may be beneficial in the treatment of other cancer types.

The epigenetic silencing of DAB2 has been investigated in other human cancers. Treatment with Trichostatin A (TSA), a well-known histone deacetylase (HDAC) inhibitor, was able to restore DAB2 expression in nasopharyngeal carcinoma, squamous cell carcinoma, and transitional carcinoma cells [47], indicating that HDAC-mediated chromatin modulation may play a role in DAB2 downregulation [48]. SCFAs may be able to recover expression of epigenetically silenced genes in several ways. SCFAs may block HBx itself thus preventing it from mediating its alterations, as butyrate has been shown to block HBx expression by inhibiting the HDAC, SIRT-1 [36]. Butyrate has also been shown to inhibit the expression of HBx in HepG2.2.15 cells [36], which has also been observed in vivo (Fig. 5). At the molecular level, SCFAs may render HDACs ineffective by competitively binding to their active sites [15], thus preventing the silencing of tumor suppressors. Decreased HBx expression in the liver of SCFA treated mice (Fig. 5) may also restore the activity of the tumor suppressor p53, the latter of which is also known to be inhibited by HBx [49]. In addition, DAB2 was identified as a target of miR-106b in cervical cancer [50]. DAB2 suppression in HCC [51] may be due to the increased expression of miR-106b, which is promoted by HBx [52]. Butyrate decreased the expression of miR-106b which was accompanied by decreased cell proliferation [53]. Thus, the targeting of HBx functions through use of SCFAs may likely provide a novel, nontoxic approach for the treatment of HBV carriers with CLD who are at high risk for the development of cirrhosis and HCC.

## Author contributions and acknowledgments

Marcia M. Clayton: resources (animal colony), Dr. Salim Merali: validation, investigation and formal analysis (contributed to the proteomics work), Carmen Merali: performed the proteomics experiments. Dae-Yeul Yu: resource (provided the HBx transgenic mice). Noreen McBrearty: visualization, writing original draft, investigation, data analysis, curation and validation as partial fulfillment for her Ph.D. degree. Dr. Alla Arzumanyan: conceptualization, methodology, and formal analysis of data Daily supervision for Noreen McBrearty. Dr. Eugene Bichenkov investigation and data curation involving tail snip analysis. Dr. Mark Feitelson: project administration, conceptualization, visualization, supervision, funding and writing (review and editing) this manuscript.

## Appendix Supplementary materials

Image, application 1
