# Supplementary material for: Short chain fatty acids delay the development of hepatocellular carcinoma in HBx transgenic mice
Source: Neoplasia. 2021 May 1;23(5):529–38. doi: 10.1016/j.neo.2021.04.004 (PMC8111251; doi:10.1016/j.neo.2021.04.004)
Supplement: Supplementary file 1 [file mmc1.docx]

**Supporting Information**

*Mice and Treatments*

Controls were sibling littermates. All mice were fed the same diet (5062, Lab Supplytx, Dallas Tx) and given water ad libitum. Mice were not fasted before treatment. Treatment was delivered during the light cycle at approximately the same time each day. Mice were housed in autoclaved cages with Bed-o’cob and domes for enrichment. After three months of treatment, mice were anesthetized with a ketamine/xylazine cocktail and then perfused with PBS.

*Immunohistochemistry*

Livers from HBxTg mice were removed, fixed in formalin, and embedded in paraffin. Five-micron thick tissue slices were prepared from these paraffin blocks. For immunohistochemistry (IHC), slides were deparaffinized, dehydrated, incubated for 30 minutes in Unitrieve antigen retrieval solution (NB325, Innovex, Richmond, CA) heated to 60°C, and stained using the UltraVision detection system (TP-015-HD, Thermo Scientific, Philadelphia, PA). Normal mouse immunoglobulin G (IgG, Santa Cruz Biotechnology, Dallas, TX) was used as a control for anti-DAB2 while pre-bleed rabbit serum from the same animal immunized to produce anti-99 (anti-HBx peptide antibodies) [27] was used as a control for HBx IHC. For DAB2 staining, rabbit polyclonal antibodies (ab76253 Abcam) were used and normal rabbit serum (pre-immune serum) was used as control. Antibody dilutions were used as recommended by the manufacturer. IHC results were recorded as + (< 20% positive cells), ++ (20-70% positive cells), and +++ (> 70% positive cells). IHC was also evaluated at the cellular level as scattered, (individual cells positive), lobular (groups of positive cells), or diffuse (most cells in a section positive). Subcellular localization for IHC was also assessed as membranous (M), nuclear (N) or cytoplasmic (C). Liver histopathology was evaluated using hematoxylin and eosin (7502, Fisher Scientific, Philadelphia, PA) staining. Slides were evaluated independently by two investigators.

*SDS/PAGE and Western blotting*

Previously snap frozen liver tissues were homogenized in lysis buffer (ab179835, Abcam) with protease inhibitor cocktail (ab65621, Abcam). Cell debris was removed by centrifugation twice at 14,000g for 15 minutes. Protein extracts from cells were prepared using the same lysis buffer. For western blotting (WB), 100 μg of protein extracts from liver tissues were separated by SDS-polyacrylamide gel electrophoresis and transferred to nitrocellulose membranes (GE10600118, GE Healthcare Life Sciences, Marlborough, MA). Membranes were incubated with anti-DAB2 (ab256524 Abcam) or anti-Shoc2 (ab106430 Abcam), and anti-β-actin (A1978 Sigma, St. Louis, MO). The blots were developed using the Odyssey western blotting kit (926-32083, Li-Cor, Lincoln, NE). Secondary antibodies were IRDye goat anti-mouse (926-32210, Li-Cor) for β-actin detection or goat anti-rabbit IgG (926-32211, Li-Cor) for DAB2 and Shoc2. Visualization was performed by OdysseyFc imaging system and quantification by Image studio 5x software ([www.licor.com](http://www.licor.com)).

*RAS Activity Assay*

A Ras activation assay (Abcam) was performed on control and SCFA-treated HBxTg liver samples to isolate GTP bound Ras according to manufacturer’s instructions. Once isolated, the samples were separated by SDS-PAGE and transferred to nitrocellulose membranes (GE Healthcare Life Sciences). Membranes were incubated with anti-ras (3965, Cell Signaling, Danvers, MA) and secondary antibody IRDye goat anti-rabbit IgG (926-32211, Li-Cor, Lincoln, NE). The blots were developed using the Odyssey western blotting kit (Li-Cor). Blots were visualized using OdysseyFc imaging system and quantified by Image studio.

*Proteomics*

Samples were centrifuged at 14,000g for 10 minutes. After protein concentration was determined using the Bradford assay (5000002 Bio-Rad), 100μg protein was digested with trypsin (rtrypsin 1.06301 Sigma-Aldrich). Samples were fractionated and desalted according to in-StageTip processing protocol as previously described [34].

Label-free proteomic analysis was performed independently on these fractions from each mouse in each group. Peptide mixtures were fractionated by ion exchange chromatography and then identified by Q exactive mass spectroscopy (Thermo Scientific) using MaxQuant software (v1.6.2.3) and further analyzed by Panther. Electrospray ionization (ESI) was delivered with an emitter (ID 30 μM, 40 mm length) at a spray voltage of −1800 V. MS/MS fragmentation was performed on the ten most abundant ions in each spectrum using collision-induced dissociation with dynamic exclusion (excluded for 10.0 s after one spectrum), with automatic switching between MS and MS/MS modes. The peptide false discovery rate (FDR) was 0.01, protein FDR 0.01, minimum peptide length was 7 amino acids, and the minimum razor and unique peptides was: 1, min. The generated peak list was processed through the Andromeda software and searched against the SwissProt mouse database (release 2018_01; 16,950 sequences). Andromeda search parameters were set as Mus musculus (species); trypsin (enzyme); carbamidomethyl (fixed modification) on Cys; (variable modification), methionine oxidation and acetyl (protein N-term); 7 ppm mass tolerance for precursor peptide ions and 20 ppm mass tolerance for product ions. Data were filtered at 1% protein and peptide spectrum matches (PSMs) FDR.

*Statistics*

Chi square analysis was used to determine significance between the percentage of treated vs control HBxTg mouse livers that developed dysplasia and HCC. The Student’s t test was used to evaluate significance in dysplastic nodule development in 9-month old treated vs control HBxTg mice as well as differences in the number of tumor nodules in 12-month old treated vs control HBxTg mice. The difference in staining intensity of DAB2 between treated vs controls was evaluated by chi square analysis. The difference in cell viability in cell lines was determined by Student’s t test. Statistical significance was considered when *P* < 0.05.

**A**

Relative intensity (%)

100

80

60

40

20

0


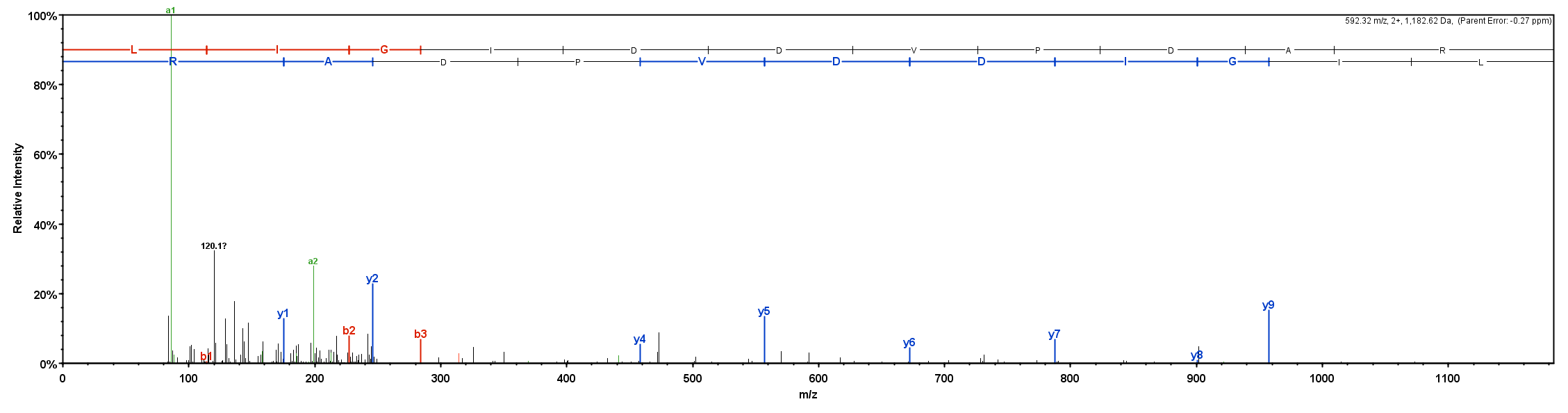


0 100 200 300 400 500 600 700 800 900 1000 1100

m/z

**B**

Relative intensity (%)

100

75

50

25

0


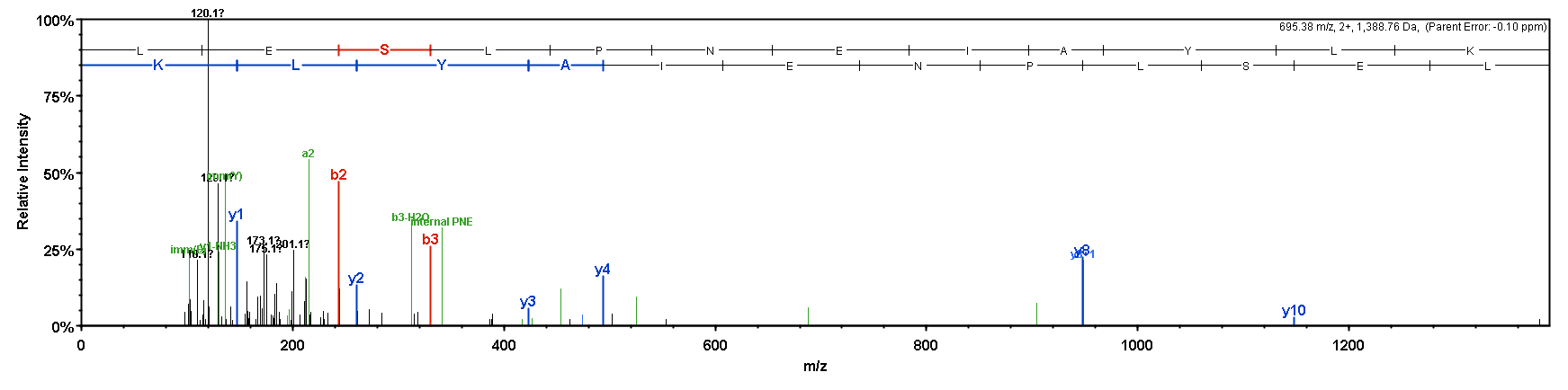


0 200 400 600 800 1000 1200

m/z

**C**

100

75

50

25

0


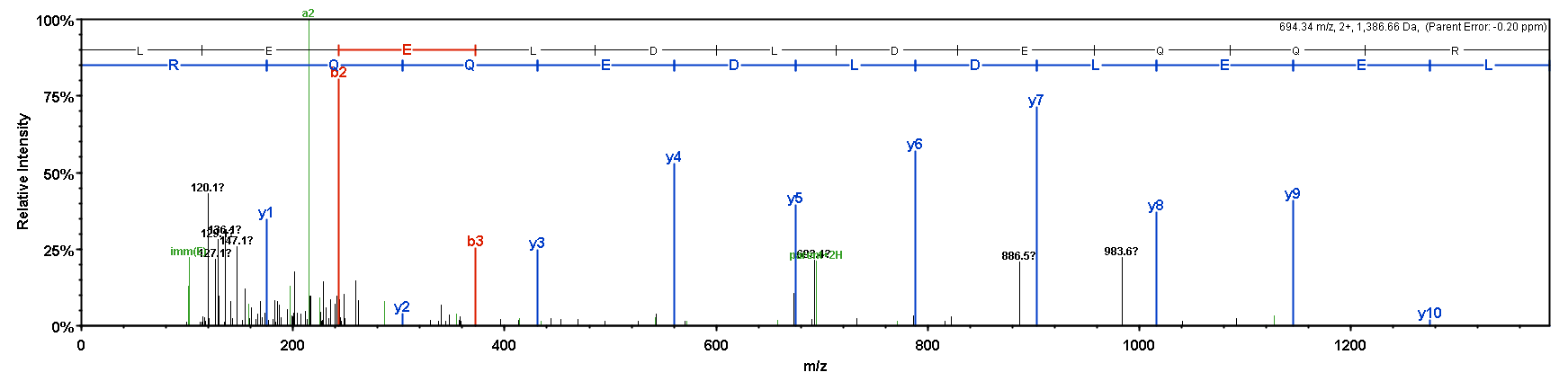


Relative intensity (%)

0 200 400 600 800 1000 1200

m/z

**Fig. S1.** (A) Disabled homolog 2 (DAB2) mass spectrum sequence identified with Maxquant with integrated Andromeda search engine; sequence: LIGIDDVPDAR, Charge +2, Monoisotopic m/z: 592.32 Da, MH+: 1182.62 Da. (B) Leucine-rich repeat protein Shoc2 mass spectrum sequence identified with Maxquant and integrated Andromeda search engine; sequence: LESLPNEIAYLK, Charge +2, Monoisotopic m/z 695.38 Da, MH+: 1388.76 Da. (C) Dual specificity mitogen-activated protein kinase kinase 2 mass spectrum sequence identified with Maxquant and integrated Andromeda search engine; sequence LEELDLDEQQR, Charge +2, Monoisotopic m/z 694.34 Da, MH+ 1386.66 Da.
